# Where does residents' choice of primary medical treatment come from?—A logical analysis based on the perspective of service accessibility and residents' cognition

**DOI:** 10.3389/fpubh.2022.949622

**Published:** 2022-10-17

**Authors:** Fang Wu, Ning Wang, Yingna Qu

**Affiliations:** School of International Pharmaceutical Business, China Pharmaceutical University, Nanjing, China

**Keywords:** primary medical treatment, service accessibility, residents' cognition, logistic, mediating effect

## Abstract

The uneven distribution of medical and health resources leads to changes in the choice of patients for medical treatment, which is the key to restrict the reform of medical services in China currently. Taking service accessibility and residents' cognition as the starting point, this study utilized the data from the questionnaire and applied logistic regression and mediation test. By taking service accessibility as an explanatory variable and residents' cognition as an intermediary variable, the study examined the differences between residents' choice of medical treatment at the primary and non-primary levels. Thus, the influencing factors of residents' choice of medical treatment at the primary level were explored. The research statistics came from questionnaires of 1,589 residents in Nanjing, Jiangsu Province, China. The results showed that service accessibility and residents' cognition were significantly correlated with the residents' choice of primary medical treatment. Household registration, age, the signing situation with family doctors, hospital service fees, and distance to the hospital were positively related to residents' choice of primary medical treatment; while the reputation, scale, residents' income, and the reimbursement ratio of residents' medical insurance were negatively correlated with the choice. In addition, residents' cognition played an intermediary effect between service accessibility and the residents' choice of primary medical treatment. The signing situation with family doctors indirectly affected the choice of primary medical treatment through residents' cognition, and residents' cognition masked some negative influence of the reimbursement ratio of residents' medical insurance on the choice of primary medical treatment.

## Introduction

The World Health Assembly first proposed the concept of “primary health care,” organized by the World Health Organization and the United Nations International Children's Emergency Fund in 1978, which was recognized as the strategic goal and basic approach to achieve that health services are available for all people by the year 2000 (1). Primary health care was regarded as an important measure to solve the waste of medical resources and promote the equalization of medical services (2). Many countries such as the United Kingdom, Canada, and Norway took primary medical institutions as the core and hospitals as the auxiliary to provide qualified and affordable medical service (3–5). In China, focusing on “first diagnosis at the primary level,” the hierarchical medical system is not only an important part of China's medical service reform but also an inevitable choice to meet the growing health needs of residents and achieve the strategic goal of “Healthy China.”

**Figure 1:**
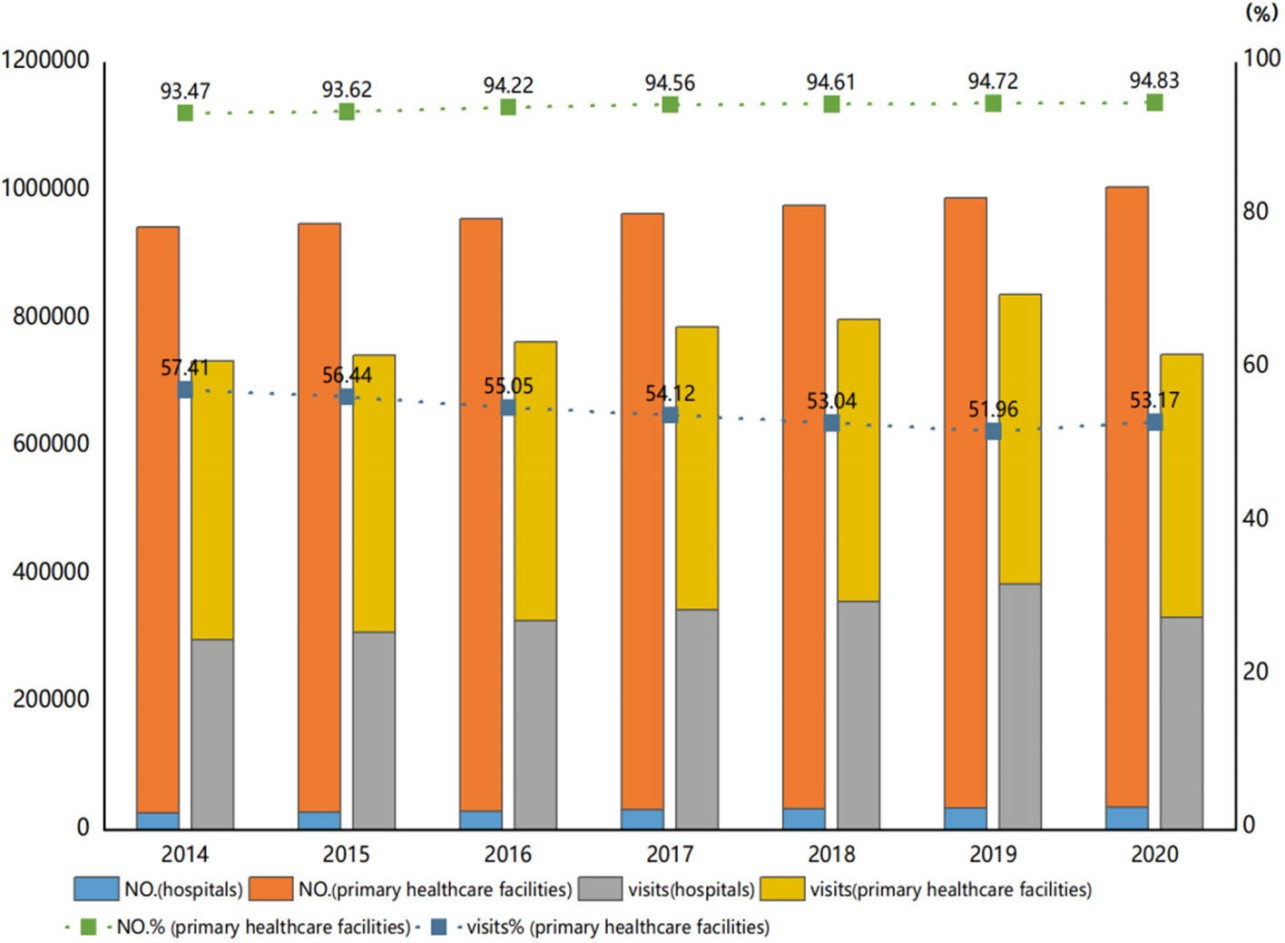
The number of medical institutions and the number of visits in China from 2014 to 2020.

In 2021, China's “Notice of the General Office of the State Council on Printing and Distributing the Key Tasks for Deepening the Reform of the Medical and Health System in 2021” and the “Medical Security Plan for the Period of 14th Five-Year” both proposed to accelerate the construction of the hierarchical medical system and carry out a pilot project of a high-quality and efficient integrated medical system. Since the issuance of the “Guiding Opinions on Promoting the Construction of hierarchical Medical Treatment System” in 2015, China has made certain improvements in the primary medical environment through the introduction of advanced medical equipment, the construction of a team of general practitioners, and the implementation of the family doctor contract system. From 2014 to 2020, the number of primary medical institutions in China increased from 917,335 to 970,036, and the number of visits to primary medical institutions increased from 297,207 to 332,288. However, the proportion of visits to primary medical institutions in China decreased from 57.41% in 2014 to 53.17% in 2020, showing a downward trend (Figure 1) (6). It can be seen that there is still great resistance to the promotion of hierarchical diagnosis and treatment in China, and the goal of diverting patients from hospitals to primary medical institutions has not been fully achieved, and residents still tend to gather at the top level of medical treatment. China's hierarchical diagnosis and treatment focus on promoting the functional positioning of various medical institutions at all levels such as provinces, cities, counties, townships, and villages and playing a balanced role. Exploring the reasons for residents' choice of medical treatment at the primary level is to promote the implementation of this system and realize the breakthrough of the sinking of high-quality medical resources (7). Therefore, under the inherent problems and new situation, how to guide residents to choose medical treatment at the primary level, promote the development of hierarchical diagnosis and treatment, and realize the sinking of high-quality medical resources have become a hot topic of the current Chinese society.

The existing research on residents' medical treatment mostly focuses on the National Health Service System, spatial and geographical location, and so on. Previous studies have shown that residents' medical treatment is not only influenced by primary health care, patient referral, general practitioner service, and other related systems in the national health service system (8, 9) but is also closely related to traffic distribution, regional spatial characteristics, and spatial coverage of medical network in spatial geographical location (10–12). However, few scholars have explored the influencing factors of primary-level medical treatment choices from the perspective of residents at the microlevel, taking patients as the center. Whether the health services are accessibly affect the choices of residents to seek medical treatment, which means that how to guide residents to seek medical treatment at the primary level depends on the service accessibility. Service accessibility refers to the process by which residents “enter the health service system by any means and choose continuous treatment” (13). Service accessibility connects the health service system and the service population, affecting residents' cognition of medical services, and it is also a major challenge for planners and decision-makers to guide residents' medical choices (14). In addition, residents' medical treatment behavior is the explicit form of residents' psychological activities and is closely related to residents' cognition. Residents' cognition determines whether a person will adopt certain medical treatment behavior intentions, and intention ultimately determines whether a certain medical treatment behavior is adopted by a person (15). Therefore, residents' cognition is indispensable for service acquisition and plays an important role in the relationship between service accessibility and choices of medical treatment. Based on this, this article attempts to study the impact mechanism of service accessibility on residents' choices of medical treatment under different scenarios. At the same time, using residents' cognition as a mediating variable, this article examined the changes in residents' choices for medical treatment and deeply analyzed the difference between residents' behavior of choosing at the primary and non-primary levels. Then, the study further explored where the choice of primary medical treatment comes to guide residents to select reasonable medical institutions for treatment, promote the advancement and implementation of hierarchical diagnosis and treatment, and realize the sinking of high-quality medical and health resources.

## Materials and methods

### Theoretical analysis and research design

#### Characteristics of residents and medical institutions

Different countries have different medical service models, but most of them maintain a structure with a clear division of labor in the medical service system centered on medical institutions (2). Residents' choices of medical treatment involve the interests of residents and medical institutions. There are differences in the choices with different sociological demographic characteristics. The services of medical institutions also affect residents' choices of medical treatment. Gender, household registration, age, and educational level are the focal factors in the study of residents' choices for medical treatment (16, 17). In addition, health resources and soft power of medical institutions are also important trade-off factors for residents to choose a doctor (18). Based on this, this article established the following hypothesis:

H1a: The individual traits of residents are significantly correlated with the choices of medical treatment.

H1b: The characteristics of medical institutions are significantly correlated with residents' choices of medical treatment.

#### Service accessibility and choices of medical treatment

Penchansky and Thomas proposed the concept of accessibility of “fit between customer and service system” in 1981 and constructed a “five-dimensional measurement method of public health care access:” Availability, Accessibility, Accommodation, Affordability, and Acceptability (19). On this basis, Peters et al. subdivided the dimensions of measuring accessibility into availability (supply of medical services), financial accessibility (price of services and resources of users), acceptability (attitudes and acceptance of users), and geographic accessibility (20). According to Penchansky and David's definition and measurement of access, this article divided service accessibility into demand-side service accessibility and supply-side service accessibility from the perspective of “supply” and “demand.” The demand-side service accessibility is that the ability of residents to obtain services provided by medical institutions involves suitability, affordability, and acceptability, that is, resource acceptance of demanders. Supply-side service accessibility refers to the health resource services provided by medical institutions to residents, involving availability resources, that is, the service cost and geographical accessibility of providers. Residents' choices for medical treatment are the external manifestation of service accessibility, and service accessibility affects residents' choices for medical treatment (21). Based on this, this article proposes the following hypothesis:

H2: Service accessibility is significantly related to the choices of medical treatment. Residents tend to choose medical institutions with higher service accessibility for treatment.
H2a: Demand-side service accessibility affects residents' choices of medical treatment. Residents will choose medical institutions with high demand-side service accessibility for treatment.
H2b: Supply-side service accessibility affects residents' choices of medical treatment, and residents will choose medical institutions with high supply-side service accessibility for treatment.

#### Service accessibility, residents' cognition, and residents' choices of medical treatment

Cognition is an important perspective for health behavior research (22). Residents' cognition represents residents' understanding of the health service information input or output by medical institutions (23). Residents' cognition is an important pivot between service accessibility and residents' choices of medical treatment. According to Fishbein's rational behavior theory for explaining and predicting individual behavior, personal attitudes and values determine whether a person will take certain behaviors, and intentions ultimately determine whether a certain behavior is adopted by a person (24). On the one hand, service accessibility also affects residents' cognition of health services to a certain extent, and out-of-service accessibility will lead to residents' lack of cognition of health services. On the other hand, residents' cognition is significantly related to their choices of medical treatment (25). By means of extensive and in-depth medical education and publicity, residents' cognition of health services can be increased and their choices of medical treatment can be influenced (26). Based on these theories, the study proposed to establish hypothesis H3.

H3: Residents' cognition has a mediating effect between service accessibility and residents' choices of medical treatment.

**Figure 2:**
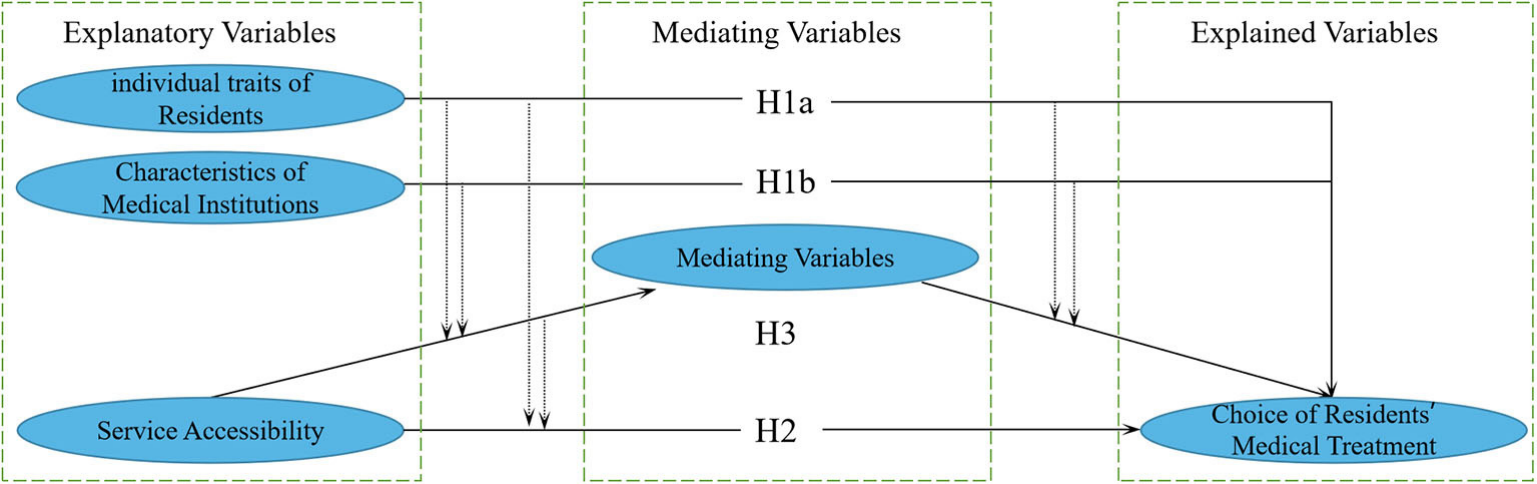
Hypothetical framework of the research on residents' choices of medical treatment.

According to the above research hypothetical, the initial relationship of the variables is shown in Figure 2.

### Data and methods

#### Data resource

Based on the statistics on the number and distribution of tertiary hospitals, specialized hospitals, and primary health service institutions in Nanjing, Jiangsu Province, China, the study further conducted a stratified sampling of hospitals and primary medical institutions in different regions according to a fixed proportion. Then, according to the distribution of hospitals and primary medical institutions, the research team went to the vicinity of hospitals and primary medical institutions from 2019 to 2020 to distribute questionnaires face-to-face. Questionnaire filling includes electronic and paper questionnaires. Recruitment criteria for volunteers included: (1) age > 18; (2) Local residents (residence > 6 months); and (3) Volunteer to participate in this study. A total of 1,802 questionnaires were distributed in this study, and 1,589 valid questionnaires were collected with an effective recovery rate of 88.18%.

#### Variable selection

Based on the research hypothesis, combined with the questionnaire, the specific assignments of variables in this article are shown in Table 1. Some of the variables used the Likert five-point scale method, and the satisfaction evaluation was set from options of very dissatisfied to very satisfied, with 1–5 points, respectively.

**Table 1:** Explanatory variables and assignments.

**Variables**	**Meanings**	**Assignments**
Individual traits of residents	Gender	Male = 1, Female = 2
	Household registration	City = 1, Rural Areas = 0
	Age	(18, 30] Years = 1, (30, 45] Years = 2, (45,60] Years = 3, More than 60 Years = 4
	Educational level	Junior high school students and below = 1, High school students = 2, College students = 3, Undergraduate students = 4
Characteristics of medical institutions	Waiting time for treatment	Measured by five-point scale (1–5) according to the objective evaluation of residents
	Reputation	
	Scale	
	Advanced degree of equipment	
Service accessibility	Income	Measured by per capita monthly income (0, 2,000] yuan = 1, (2,000, 3,000] yuan = 2, (3,000, 4,000] yuan = 3, (4,000, 5,000] yuan = 4, Above 5,000 yuan = 5
	Reimbursement ratio of residents' medical insurance	Measured by five-point scale (1–5) according to the objective evaluation of residents
	Signing situation with family doctors	
	Hospital service fees	
	Distance to the hospital	
Mediating variables	Residents' cognition	Measured by five-point scale (1–5) according to the objective evaluation of residents
Dependent variable	Residents' choices of medical treatment	Non-primary medical institutions = 0 Primary medical institutions = 1

##### Explained variables

The explained variable is residents' choice of medical treatment. Regarding the medical institutions selected for treatment, the study divides medical institutions into primary medical institutions and non-primary medical institutions according to China's “National Medical and Health Service System Planning Outline (2015–2020)” and “2021 China Health Statistical Yearbook.” Among them, primary medical institutions mainly include township health centers, community health service centers (stations), village clinics, infirmaries, outpatient departments (offices), and military primary health institutions. Non-primary medical institutions include hospitals, nursing homes, and professional public health institutions (6, 27).

##### Core explanatory variables

According to the research hypothesis, combined with the questionnaire, this article selected service accessibility as the core explanatory variable. Service accessibility was divided into demand-side accessibility and supply-side accessibility. Demand-side service accessibility was from the perspective of residents' needs and could be expressed by factors such as income, reimbursement ratio of residents' medical insurance, and the signing situation with family doctors (5, 28, 29). Supply-side service accessibility was from the perspective of the supply of medical institutions, including hospital service fees, distances to hospital, and other factors (30, 31). In this study, the reimbursement ratio of residents' medical insurance refers to the overall reimbursement ratio of medical institutions, including primary medical institutions and non-primary medical institutions. Its measurement is based on residents' evaluation of the reimbursement ratio of current medical institutions.

##### Explanatory variables

In this article, the individual traits of residents and the characteristics of medical institutions were used as explanatory variables. The individual traits of residents were selected as gender, household registration, age, and educational level. The characteristics of medical institutions were selected as the waiting time, reputation, scale, and advanced degree of equipment. In this study, the characteristics of medical institutions were scored based on residents' perceptions of the importance of these elements.

##### Mediating variables

According to hypothesis H3, the study used residents' cognition as a mediating variable. Residents' cognition is measured from high to low by using Likert five-scale method based on residents' understanding of the content and policies of primary medical and health services, which mainly include cognition of primary medical health service and cognition of relevant policies (32, 33).

#### Statistical analysis

All statistical analyses were performed based on spss26.0 software. Firstly, descriptive analysis was used to present the basic situation of the sample objects through frequency, percentage, mean, and standard deviation. Subsequently, reliability and validity tests and collinearity analysis were conducted on the data. Based on this, the stepwise regression analysis was adopted, and the logistic regression model was constructed to explore the influencing factors of residents' choice of primary medical treatment. Finally, with reference to the test method of mediating effect, the association between residents' perception of access to intervention services and residents' choice of primary medical treatment was further analyzed. Based on the Sobel method, 95% confidence interval, Z-statistic, and effect value were obtained to further test the mediating effect of residents' cognition (34, 35). *P* < 0.05 was statistically significant in this study.

#### Model settings

##### Service accessibility and residents' choices of medical treatment

To test the hypotheses H1a, H1b, and H2, the following models were constructed:


(1) $$\begin{array}{l} logit\left(Y\right)=\beta_{1}Resident+\beta_{2}Institution+\beta_{3}Service+\varepsilon \end{array}$$

Formula (1) was the logistic regression model, and Y was the dependent variable, indicating the residents' choices of medical treatment. *Resident* represented the individual traits of residents, including gender, household registration, age, and educational level. *Institution* represented the characteristics of medical institutions, including waiting time, reputation, scale of medical institutions, and advanced degree of equipment. *Service* indicated service accessibility, including residents' income, resident reimbursement ratio of residents' medical insurance, signing situation with family doctors, hospital service fees, and distance to the hospital. In the formula, β_1_,β_2_,β_3_ represented the regression coefficient, and **ε** represented the error term.

##### Service accessibility and residents' choices of medical treatment on both supply-side and demand-side

To test Hypothesis H2a and Hypothesis H2b, the following models were constructed:


(2) $$\begin{array}{l} {logit}_{1}\left(Y\right)=\lambda_{1}{Resident}_{j}+\lambda_{2}{Institution}_{j}+\lambda_{3}{Service}_{1}+\varepsilon_{1}\\ {logit}_{2}\left(Y\right)=\alpha_{1}{Resident}_{j}+\alpha_{2}{Institution}_{j}+\alpha_{3}{Service}_{2}+\varepsilon_{2} \end{array}$$

Formula (2) was a logistic regression model, and *Resident*_*j*_ and *Institution*_*j*_ represented the variables of Resident and Institution in Formula (1) that are significantly related to residents' choices for medical treatment. *Service*_1_ indicated the service accessibility on the demand side, including residents' income, reimbursement ratio of residents' medical insurance, and signing situation with family doctors. *Service*_2_ indicated the service accessibility on the supply side, including hospital service fees, distance to the hospital. λ_1_,λ_2_,λ_3_ and α_1_,α_2_,α_3_ are the regression coefficient, ε_1_ and ε_2_ are the error term.

##### Service accessibility, residents' cognition, and choices of medical treatment


(3) $$\begin{array}{l} M=c_{1}Resident_{j}+c_{2}Institution_{j}+c_{3}Service_{j}+\varepsilon_{3} \end{array}$$

(4) $$\begin{array}{l} logit_{3}\left(Y\right)=\partial_{1}M+\partial_{2}Resident_{j}+\partial_{3}Institution_{j}\\ +\partial_{4}Service_{j}+\varepsilon_{4} \end{array}$$

Formula (3) was a multiple linear regression model, and Formula (4) was a logistic regression model. *M* was a mediating variable, which represented residents' cognition. *Service*j represented the remaining service accessibility variables in Formula (1) after excluding variables that were not related to residents' choices of medical treatment. *c*_1_,*c*_2_,*c*_3_ and ∂_1_, ∂_2_, ∂_3_, ∂_4_ were regression coefficients, and ε_3_ and ε_4_ were error terms.

## Results

### Basic information of sample objects

Among the sample subjects, only 16.4% of the people chose primary medical institutions for medical treatment, and 83.6% chose non-primary medical institutions (Table 2). Among genders, 46.9% of the people were male and 53.1% were female. As for household registration, 62.4% of the people had city household registration and 37.6% had rural one. As far as age, 44.4% of the people were younger than 30 years old, 32.8% were between the age of (30, 45], 17.6% were between (45, 60], and 5.2% were older than 60 years old. Among the educational degree, 13.9% of the people had a junior high school degree or below, 17.5% had a high school or technical secondary school degree, 18.8% had a college degree, and 49.8% had an undergraduate degree or above.

**Table 2:** Basic information of sample objects.

**Variables**		**Group**	**Assignment**	**Frequency**	**Percentage (%)**	**Average**	**Standard deviation**
Residents' choices of medical treatment		Primary medical institutions	1	261	16.4	0.16	0.37
		Non-primary medical institutions	0	1328	83.6		
Basic information of sample objects	Gender	Male	1	745	46.9	1.53	0.50
		Female	2	844	53.1		
	Household registration	City	1	991	62.4	1.38	0.49
		Rural area	0	598	37.6		
	Age	(18, 30] years	1	705	44.4	1.84	0.90
		(30, 45] years	2	521	32.8		
		(45,60] years	3	279	17.6		
		Above 60 years old	4	84	5.2		
	Degree	Junior high school degree or below	1	220	13.9	3.05	1.11
		High school or technical secondary school degree	2	278	17.5		
		College degree	3	299	18.8		
		Undergraduate degree or above	4	792	49.8		

**Figure 3:**
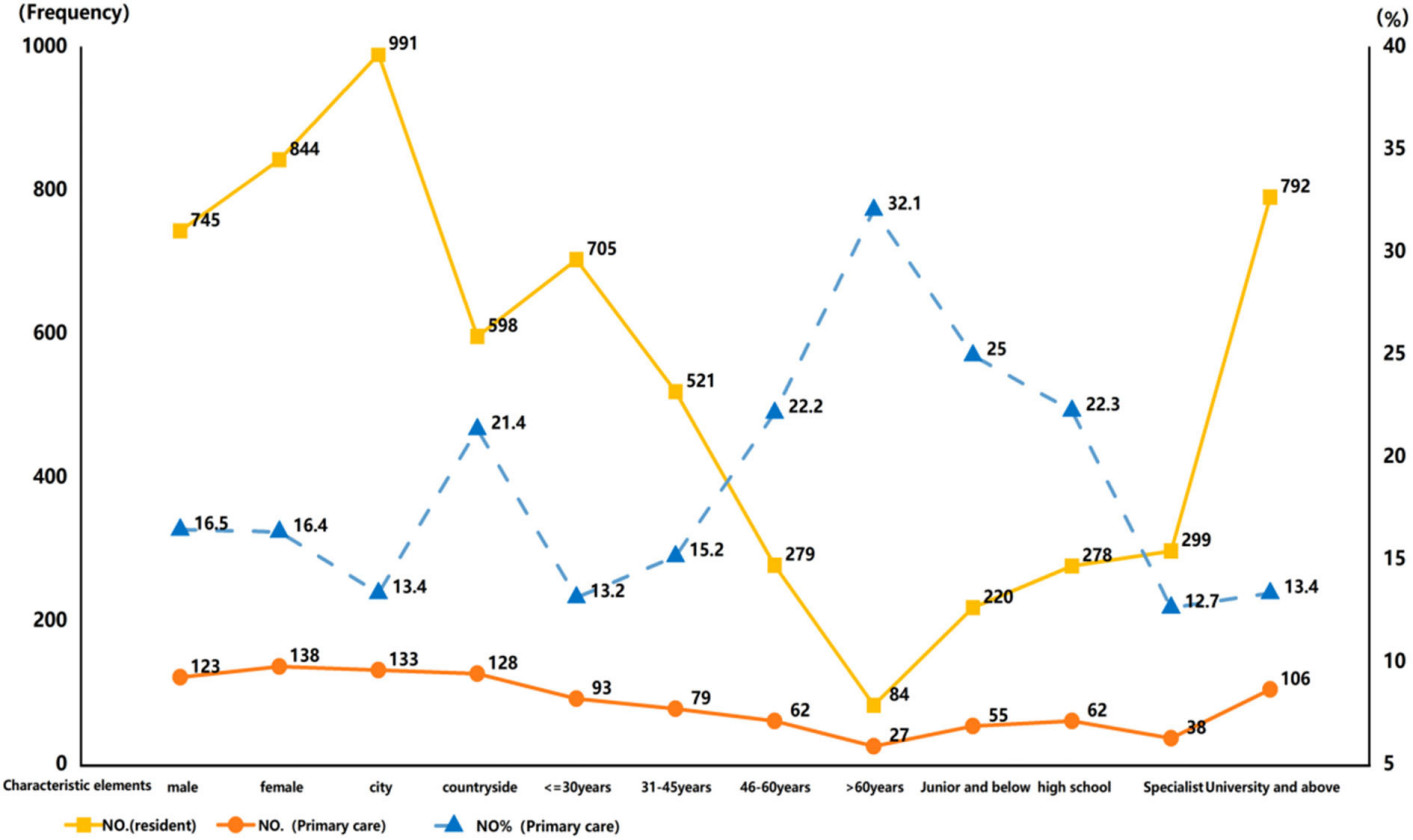
Residents' choices of medical treatment and the proportion of grassroots under the characteristic elements.

Based on the basic information of the sample subjects, further analysis of gender, household registration, age, and education level (Figure 3) showed the following percentages; 16.5% of males and 16.4% of females chose to seek medical treatment at the primary level. In terms of household registration, 13.4% of participants in city and 21.4% in rural areas selected primary medical treatment. In terms of age, 13.2% of participants below 30 years old, 15.2% between (30, 45], 22.2% between (45, 60], and 32.1% of participants above 60 years old chose primary medical treatment. In terms of education level, 25% of junior high school students and below, 22.3% of high school students, 12.7% of college students, and 13.4% of undergraduate students and above chose to seek medical treatment at the primary level. It was initially found that residents with different gender, household registration, age, and educational level had different choices for medical treatment.

### Sample data test

For the sample data from different sources, variance analysis and one-way ANOVA of variance were performed for the main variables. The results showed that there was no significant difference between the data samples from the two sources, so the data can be put together for subsequent follow-up research and analysis. The Cronbach's α coefficient of the total items in the research questionnaire was 0.921, approaching to 1, showing good reliability. In addition, the Content Validity Index (CVI) of the questionnaire was 0.94 > 0.90, and the Bartlett test of sphericity was significant at *p* < 0.05, indicating good content and construct validity. In addition, due to the problems in the setting of the research model and the data, there was likely to be a certain degree of a linear relationship between the explanatory variables of the model. Therefore, multicollinearity diagnosis was conducted for all explanatory variables in the study, and the results showed that the variance inflation factor (VIF) of all explanatory variables was <5, which met the requirement that statistical VIF shall be <10, indicating that there was no collinearity problem among explanatory variables.

### Logistics regression analysis results

In order to further explore the factors that affect residents' choices of primary medical treatment, this study conducted hierarchical regression on the three dimensions of residents' individual traits, medical institution characteristics, and service accessibility according to formula (1). The results are shown in Table 3. Model 1 only included individual characteristics of residents, including gender, household registration, age, and educational level. A statistical test showed that household registration (*p* < 0.01) and age (*p* < 0.01) had significant effects on residents' choices of medical treatment. In addition, the regression coefficient between household registration and age was larger than 0 (β > 0), that is, household registration and age were positively correlated with the choices of primary medical treatment. The estimated values of the coefficients of gender and educational level were not significant, and their relationship with residents' choices of medical treatment remains to be observed. The results showed that among the individual characteristics of residents, household registration, and age significantly affected residents' choices of medical treatment. Rural residents were more likely to choose primary medical treatment than urban residents. The older the residents were, the more they preferred primary medical institutions. Thus, hypothesis H1a was verified.

**Table 3:** Logistics regression analysis results.

**Explanatory variable**	**Model 1 (Individual traits was introduced)**		**Model 2 (Based on Model 1, the characteristics of medical institutions was introduced)**		**Model 3 (On the basis of Model 2, service accessibility was introduced)**	
	β	**OR**	β	**OR**	β	**OR**
Gender	0.001	1.001	0.013	1.013	−0.016	0.984
Household registration	0.593^***^	1.810	0.604^***^	1.830	0.598^***^	1.818
Age	0.347^***^	1.415	0.336^***^	1.400	0.298^***^	1.347
Educational level	−0.094	0.911	−0.103	0.902	−0.046	0.955
Waiting time for treatment			0.027	1.028	−0.094	0.910
Reputation			−0.170^**^	0.844	−0.198^**^	0.821
Scale of medical institutions			−0.183^**^	0.833	−0.274^***^	0.760
Advanced degree of equipment			0.101	1.107	0.062	1.064
Income					−0.121^**^	0.886
Reimbursement ratio of residents' medical insurance					−0.167^**^	0.846
Signing situation with family doctors					0.408^***^	1.503
Hospital service fees					0.226^***^	1.253
Distance to the hospital					0.218^**^	1.244

β refers to the regression coefficient, OR refers to the odds ratio;

*** and ** represent significance that are < 1 and 5%, respectively.

Based on Model 1, Model 2 incorporated the characteristics of medical institutions, including waiting time for treatment, reputation, scale of medical institutions, and advanced degree of medical equipment. The results are shown in Table 3. The regression coefficient β of reputation and medical institution was <0, which was significant at 5% level. The estimated values of waiting time and advanced degree of medical equipment were not significant, and their relationship with residents' choices of medical treatment was not yet obvious. Model 2 regression results showed that the characteristics of medical institutions were significantly correlated with residents' choices of medical treatment. Among them, the greater the reputation and scale of medical institutions attract residents to see a doctor, and the lower the probability of residents' choice for primary medical treatment. Thus, hypothesis H1b was true.

Based on Model 2, Model 3 further introduced service accessibility, including income, reimbursement ratio of residents' medical insurance, the signing situation with family doctors, hospital service fees, and distance to the hospital. In the regression results of model 3, income (*p* < 0.05, β = −0.121) and the reimbursement ratio of residents' medical insurance (*p* < 0.05, β = −0.167) were negatively correlated with the choice of primary medical treatment. The regression coefficients of the signing situation with family doctors (*p* < 0.01, β = 0.408), hospital service fees (*p* < 0.01, β = 0.226), and the distance to the hospital (*p* < 0.05, β = 0.218) were positively correlated with the choice of primary medical treatment. The results showed that service accessibility was significantly correlated with the choices of medical treatment, and residents tended to choose the medical treatment with higher service accessibility. Among them, the lower the residents' income and the proportion of medical insurance reimbursement, the better the signing situation with family doctors, the higher the hospital service fees, and the longer the distance to the hospital nearby, the higher the probability for residents choosing the primary hospital. Thus, hypothesis H2 was true.

### Regression analysis of service accessibility on supply-side and demand-side

According to the assumptions of H2a and H2b, service accessibility can be divided into demand-side service accessibility and supply-side service accessibility. The demand-side was gained from the perspective of residents, and the supply side the perspective of medical institutions. On the basis of incorporating the variables significantly related to the choices of medical treatment from residents' individual traits and medical institution characteristics, the study constructed Model 4 and Model 5 from the perspectives of service accessibility on the demand side and the supply side, respectively. The regression analysis of the two models was conducted in Formula (2), of which the results are shown in Table 4.

**Table 4:** Regression results of service accessibility on demand side and supply side.

**Explanatory variable**	**Model 4 (demand side)**		**Model 5 (supply side)**	
	**β**	**OR**	**β**	**OR**
Household registration	0.625^***^	1.869	0.661^***^	1.937
Age	0.341^***^	1.407	0.369^***^	1.446
Reputation	−0.145*	0.865	−0.215^**^	0.807
Scale of medical institutions	−0.183^**^	0.833	−0.205^**^	0.815
Income	−0.145^***^	0.865		
Reimbursement ratio of residents' medical insurance	−0.154^**^	0.857		
Signing situation with family doctors	0.399^***^	1.490		
Hospital service fees			0.218^***^	1.244
Distance to the hospital			0.187^**^	1.206

β refers to the regression coefficient, OR refers to the odds ratio;

*** and ** represent significance are <1 and 5%, respectively.

Based on household registration, age, reputation, and scale of medical institutions, Model 4 introduced the variables of income, reimbursement ratio of residents' medical insurance, and the signing situation with family doctor from the demand-side service accessibility. In the regression results of Model 4, income (β = −0.226) and the signing situation with family doctor (β = 0.399) were significant at the 1% level. The reimbursement ratio of residents' medical insurance (β = −0.154) was significant at 5% level. The results showed that demand-side service accessibility affected residents' choices of medical treatment, and residents would choose the medical institutions with high accessibility of service on the demand side. The lower the residents' income and the proportion of medical insurance reimbursement, the better the signing situation with family doctors, and the higher the probability for residents choosing primary medical institutions. Thus, hypothesis H2a was true.

Based on household registration, age, reputation, and scale of medical institutions, Model 5 incorporated two variables of distance to the hospital and hospital service fees from the demand-side service accessibility. Among them, the regression coefficients β of the hospital service fees (*p* < 0.01) and the distance to the hospital (*p* < 0.05) were all >0, which were positively correlated with residents' choice of medical treatment. The results showed that supply-side service accessibility affected residents' choices of medical treatment, and residents would choose medical institutions with high accessibility of service on the supply side. The higher the hospital service fees and the farther the distance to the hospital nearby, the greater the probability that residents would choose a primary medical institution for medical treatment. Thus, hypothesis H2b was true.

The regression results of Model 4 and Model 5 showed that, regardless of the accessibility of services on the demand-side or the supply-side, service accessibility had a significant impact on residents' choices of medical treatment. In addition, residents tended to choose the medical treatment with high service accessibility, which further verified hypothesis H2 in this article. Furthermore, the regression results showed that the signing situation with family doctors had the largest regression coefficient among the variable of service accessibility, that is, it influenced most on residents' choice of primary medical institutions.

### Analysis of the mediating effect of residents' cognitive degree

Referring to the test method of mediating effect (36), this article combined logistic regression and multiple linear regression and used residents' cognition as a mediating variable to explore the impact of service accessibility on residents' choices of medical treatment, as shown in Table 5.

**Table 5:** Testing results of mediating effect of residents' cognition degree.

**Variable**	**Model 6 (treatment choice)**	**Model 7 (residents' cognition)**	**Model 8 (treatment choice)**
Residents' cognition			0.330^***^ (0.096)
Income	−0.134^**^ (0.056)	—	−0.140^**^ (0.056)
Reimbursement ratio of residents' medical insurance	−0.177^**^ (0.078)	0.324^***^ (0.020)	−0.278^***^ (0.078)
Signing situation with family doctors	0.405^***^ (0.075)	0.514^***^ (0.019)	0.238^***^ (0.088)
Hospital service fees	0.218^***^ (0.081)	—	0.219^***^ (0.082)
Distance to the hospital	0.190^**^ (0.096)	—	0.177^**^ (0.097)
Reputation	−0.207^**^ (0.087)	—	−0.212^**^ (0.088)
Scale of medical institutions	−0.256^**^ (0.083)	—	−0.255^***^ (0.083)
Household registration	0.622^***^ (0.150)	0.097^**^ (0.040)	0.595^***^ (0.151)
Age	0.324^***^ (0.075)	0.043^**^ (0.021)	0.304^***^ (0.075)
Cox & Snell R2/R2	0.067	0.560	0.074
N	1589	1589	1589

The values are regression coefficient and standard error.

*** and ** represent significance that is <1 and 5%, respectively. The significance that is larger than or equal to 5% was not displayed.

In the first stage of mediating effect, residents' choices of medical treatment were taken as a dependent variable, and residents' individual traits, medical institution characteristics, and significant influencing factors in service accessibility in the above analysis were incorporated into the formula (1) as independent variables. Thus, Model 6 was constructed, and the test results again verified that service accessibility was significantly correlated with residents' choices of medical treatment. In the second stage of mediating effect, residents' cognition was taken as a dependent variable, and the above significant influencing factors of residents' individual traits and medical institution characteristics were brought into formula (3) as independent variables, and model 7 was constructed. The regression results showed that the signing situation with family doctors (β = 0.514, *p* < 0.01) and the reimbursement ratio of residents' medical insurance (β = 0.324, *p* < 0.01) had a significant impact on residents' cognitive degree, while other variables did not have such impact and were not considered specifically. In the third stage of mediating effect, residents' medical choices were taken as the dependent variable, residents' cognition and residents' individual traits, characteristics of medical institutions, and significant influencing factors in service accessibility were taken as independent variables in formula (4), and model 8 was constructed for regression analysis. The results showed that residents' cognition (β = 0.330, *p* < 0.01), signing situation with family doctors (β = 0.238, *p* < 0.01), and residents' medical insurance reimbursement ratio (β = −0.278, *p* < 0.01) had a significant influence on residents' choices of medical treatment. In the three stages of the intermediary test, the regression coefficients of signing situation with family doctors and reimbursement ratio of residents' medical insurance were significant in model 6, model 7, and model 8. According to the intermediary effect test conditions (37), this showed that residents' cognition played an intermediary effect between signing situation with family doctors, reimbursement ratio of residents' medical insurance, and residents' medical treatment choice. In general, the research hypothesis H3 was preliminarily validated, and residents' cognition had a mediating effect between service accessibility and medical treatment choice.

This study further explored the mediating effect of residents' cognition on the signing situation with family doctors, reimbursement ratio of residents' medical insurance, and residents' choices of medical treatment. The regression coefficients obtained from different equations are comparable only if the scales were the same, and the value of the mediating effect value can be calculated (38). Whereas, the dependent variable is a categorical variable and the independent variable, as well as the mediating variable, are both interval variables. Therefore, in this article, in Models 7, 8, and 9, residents' cognition, signing situation with family doctors, and reimbursement ratio of residents' medical insurance were standardized to achieve an equal scale of regression coefficients. Then, the Sobel method was used to verify the mediating effect of residents' cognition, and the results are shown in Table 6.

**Table 6:** Sobel mediating effect test results of residents' cognition degree.

**Path**	**Total effect**	**Mediating effect**	**Direct effect**	**Z-statistic**	**Proportion in total effect**	**Sobel 95% confidence interval**
The signing situation with family doctors → residents' cognition → choice of medical treatment	0.24	0.10	0.14	3.41	0.42	(0.042, 0.158)
Reimbursement ratio of residents' medical insurance → residents' cognition → choice of medical treatment	−0.10	0.06	−0.16	2.65	–	(0.016, 0.110)

The 95% confidence intervals in the two paths of the influence of signing situation with family doctors and reimbursement ratio of residents' medical insurance on the choice of visit do not include 0 and Z > 0.97. The results again verified that residents' cognition had a significant impact on the signing situation with family doctors, reimbursement ratio of residents' medical insurance, and residents' choice of medical treatment. Through further analysis, it was found that in the path of signing situation with family doctors → residents' cognition → choice of medical treatment, the total effect of the signing situation with family doctors on residents' choices of medical treatment was 0.24; while after incorporating residents' cognition, the direct effect was 0.14, namely the mediating effect through residents' cognition was 0.10, accounting for 42% of the total effect. In the path of reimbursement ratio of residents' medical insurance → residents' cognition → choice of medical treatment, the total effect of reimbursement ratio of residents' medical insurance on medical treatment choice was −0.10; while after incorporating residents' cognition, the direct effect of residents' reimbursement ratio of residents' medical insurance was −0.16, and the mediating effect was 0.06. Because the mediating effect and direct effect of residents' cognition on the ratio of residents' medical insurance reimbursement and residents' choice of medical treatment are opposite in sign, in this case, the mediating effect was the masking effect (39, 40).

The results showed that the signing situation with family doctors indirectly affected the choice of primary medical treatment through residents' cognition. The residents' cognition masked the negative impact of reimbursement ratio of residents' medical insurance on this choice. Specifically, the better the signing situation with family doctors was, the higher residents' cognition degree of medical institutions would be, which could promote residents' preference for primary medical institutions. To some extent, residents' cognition masked the impact of reimbursement ratio of residents' medical insurance on residents' medical primary treatment choice. That is, when the reimbursement ratio of residents' medical insurance remained unchanged, increasing residents' cognition of medical institutions could appropriately encourage residents to choose primary medical institutions, further verifying hypothesis H3.

## Discussion

From the perspective of residents' cognition and service cognition, this study explored the origin of residents' choices to seek primary medical treatment. Our results showed that the proportion of residents seeking medical treatment in primary medical institutions is low in China at the present. Uneven distribution of medical and health resources leads to changes in patients' medical choices, which was one of the typical problems in China's medical and health services (12). Therefore, we had several aspects to discuss.

First of all, we found that residents' choices of medical treatment varied with different characteristics of residents and medical institutions. In the first place, among the individual traits of residents, household registration and age significantly affected residents' choice of primary-level medical institutions, which were mainly for rural and elderly residents. In the second place, among the characteristics of medical institutions, reputation and scale significantly affected residents' choice of primary-level medical institutions. The low reputation and scale of primary-level medical institutions were important reasons for residents to choose non-primary medical institutions for treatment. Therefore, the Chinese government should further optimize the allocation of medical resources and improve the status quo that the service quality of China's primary medical institutions is still poor (41), to better utilize the “network bottom” of primary medical institutions.

Secondly, we found that service accessibility was significantly correlated with residents' choice of primary medical treatment. Service accessibility could be divided into demand-side service accessibility and supply-side service accessibility. On the one hand, demand-side service accessibility included residents' income, reimbursement ratio of residents' medical insurance, and the signing situation with family doctors. Among them, the income and reimbursement ratio of residents' medical insurance are negatively correlated with the choice of primary medical institutions. The higher the income, the lower the probability of residents choosing primary medical institutions. When the current reimbursement ratio of primary medical institutions and non-primary medical institutions increases by the same proportion, the probability of residents choosing primary medical institutions will decrease. This finding was consistent with earlier research conclusions that residents' choices of medical treatment can be affected by their own financial burden (42). The signing situation with family doctor was positively related to residents' choices of primary medical treatment. The better the signing situation with family doctors, the greater the probability that residents would choose primary medical institution. These research results are in concert with the direction of “promoting the adoption of flexible family doctor contract service cycles” mentioned in the “Notice of the General Office of the State Council on Printing and Distributing Key Tasks for Deepening the Reform of the Medical and Health System in 2021.” On the other hand, supply-side service accessibility covered hospital service fees and distance to hospital. Among them, hospital service fees were positively related to residents' choices of primary medical treatment. The higher the hospital service fees, the higher the probability of residents choosing primary medical institutions. This was consistent with the conclusion of an earlier study on the choice of primary care for residents in Beijing, China (43). Distance to hospital was positively related to residents' choices of primary medical treatment. The longer the distance to the hospital, the greater the probability that residents choose primary medical institutions for medical treatment, that is, the convenience of access to services was an important reason for residents to choose primary medical institutions for medical treatment. This was consistent with the conclusion of earlier scholars' research on the influencing factors of residents' choice of primary medical treatment in Southwest China (44). Therefore, on the one hand, the Chinese government should encourage the development of new medical service mode, such as chain clinics, Internet medical services, and doctor groups to reasonably improve the layout of medical institutions. On the other hand, flexible signing with family doctors can be adopted to explore the binding signing service between medical insurance and family doctors. Under this binding mode, family doctors can obtain income from the medical insurance balance of contracted patients for incentive, so as to realize the full coverage of signing with family doctors.

Finally, we found that residents' cognition had an important impact on residents' choice of primary medical treatment and played a mediating effect between service accessibility and medical treatment choice. Among these, the signing situation with family doctors indirectly affected the choice of primary medical treatment through residents' cognition, and residents' cognition masked some negative influence of the reimbursement ratio of residents' medical insurance on the choice of primary medical treatment. Specifically, the impact of signing situations with family doctors on residents' choices of medical treatment was partly realized through residents' cognition. By enhancing residents' cognition, the proportion of residents who choose primary medical institutions for medical treatment can be increased; Due to the policy provisions, it is difficult to adjust the reimbursement ratio of residents' medical insurance in a certain period of time. Residents' cognition can mask the negative impact of some residents' medical insurance reimbursement proportion on the choice of primary medical treatment. These findings were consistent with the research conclusions of early scholars that the residents' cognition can reduce the inaccessibility and inequality in health services (21), which was an effective way to improve residents' medical choice (45). Therefore, the Chinese government and medical institutions can enhance residents' cognition, widely popularize health education for residents, regularly carry out routine examination and chronic disease management, integrate the concept of whole life-cycle health management, and implement people-oriented integrated medical services, to indirectly guide residents' choice of primary medical treatment.

We acknowledge that there are still some limitations in the study. First of all, there may be some undetected confounding factors in our study, such as external factors of decision-making by others, unbalanced development of specialized medical care, and social relationships. In addition, in future research, if we want to explore the primary medical options of more specific group, further evaluation and observation should be conducted for different groups of residents.

## Conclusion

Our study aims to explore the influencing factors of residents' choice of primary medical institutions, which is of great significance for China's current medical reform. Based on the data of the questionnaire for residents in Nanjing, Jiangsu Province, logistic regression and mediation test were used to find that there was a significant correlation between service accessibility, residents' cognition, and the residents' choice of primary medical treatment. Among them, household registration, age, signing situation with family doctors, hospital service fees, and distance to the hospital were positively correlated with the residents' choice of primary medical treatment; the reputation, scale, income, and reimbursement ratio of residents' medical insurance were negatively correlated with the residents' choice of primary medical treatment. In addition, the study also found that residents' cognition played a mediating effect between service accessibility and residents' choice of primary medical treatment. The signing situation with family doctors indirectly affected the residents' choice of primary medical treatment through residents' cognition, and residents' cognition masked some negative influence of the reimbursement ratio of residents' medical insurance on the residents' choice of primary medical treatment. The Chinese government should optimize the allocation of medical resources, taking both the service accessibility and residents' cognition into account, to promote the advancement and implementation of a hierarchical medical system and realize the sinking of medical and health resources of high quality. Specifically, first, the Chinese government can adopt flexible family doctor contract and explore the bundling of medical insurance and family doctor contract services. Thus, family doctors can obtain partial income from the medical insurance balance of contracted patients as incentives to achieve full coverage of family doctor contracts and improve service accessibility. Second, primary medical institutions can cooperate with hospitals through medical treatment combination, medical groups, etc. On the one hand, health education, chronic disease prevention, and other health services can be carried out; on the other hand, free clinics and other activities centered on patients can be developed regularly. Therefore, residents' cognition of the content and policies of primary medical and health services can be improved, and information asymmetry between medical institutions and residents can be reduced. Ultimately, it is expected to promote the development and implementation of graded diagnosis and treatment in China and realize the sinking of high-quality medical and health resources.

## Data availability statement

The original contributions presented in the study are included in the article/supplementary files, further inquiries can be directed to the corresponding author/s.

## Author contributions

FW and NW: conceptualization, data curation, methodology, writing—original draft preparation, and funding acquisition. FW and YQ: writing—review and editing. All authors have read and agreed to the published version of the manuscript.

## Funding

This study was funded by Humanity and Social Science Youth Foundation of Ministry of Education of China (19YJC630183), the Key Project of Social Science Foundation in Jiangsu Province (20ZLA014), and the General Project of Philosophy and Social Science Research in Colleges and Universities in Jiangsu Province (2019SJA0058).

## Conflict of interest

The authors declare that the research was conducted in the absence of any commercial or financial relationships that could be construed as a potential conflict of interest.

## Publisher's note

All claims expressed in this article are solely those of the authors and do not necessarily represent those of their affiliated organizations, or those of the publisher, the editors and the reviewers. Any product that may be evaluated in this article, or claim that may be made by its manufacturer, is not guaranteed or endorsed by the publisher.
